# Supplementary material for: Molecular mechanism of a covalent allosteric inhibitor of SUMO E1 activating enzyme
Source: Nat Commun. 2018 Dec 4;9:5145. doi: 10.1038/s41467-018-07015-1 (PMC6279746; doi:10.1038/s41467-018-07015-1)

Supplementary Information

Molecular mechanism of a covalent allosteric inhibitor of SUMO E1 activating enzyme

Zongyang Lv[1,4], Lingmin Yuan[1,4], James H. Atkison[1], Katelyn M. Willams[1], Ramir Vega[2], E. Hampton Sessions[3], Daniela B. Divlianska[3], Christopher Davies[1], Yuan Chen[2*], & Shaun K. Olsen[1*]

[1]Department of Biochemistry & Molecular Biology and Hollings Cancer Center, Medical University of South Carolina, Charleston, South Carolina, USA

[2]Department of Molecular Medicine, Beckman Research Institute of City of Hope, Duarte, California, USA

[3]Conrad Prebys Center for Chemical Genomics, Sanford Burnham Prebys Medical Discovery Institute at Lake Nona, Orlando, Florida, USA

[4]These authors contributed equally to this work

* Correspondence should be addressed to Y.C. (ychen@coh.org) and S.K.O. (olsensk@musc.edu)

Supplementary Information Inventory:

Supplementary Figures 1-6
Supplementary Note

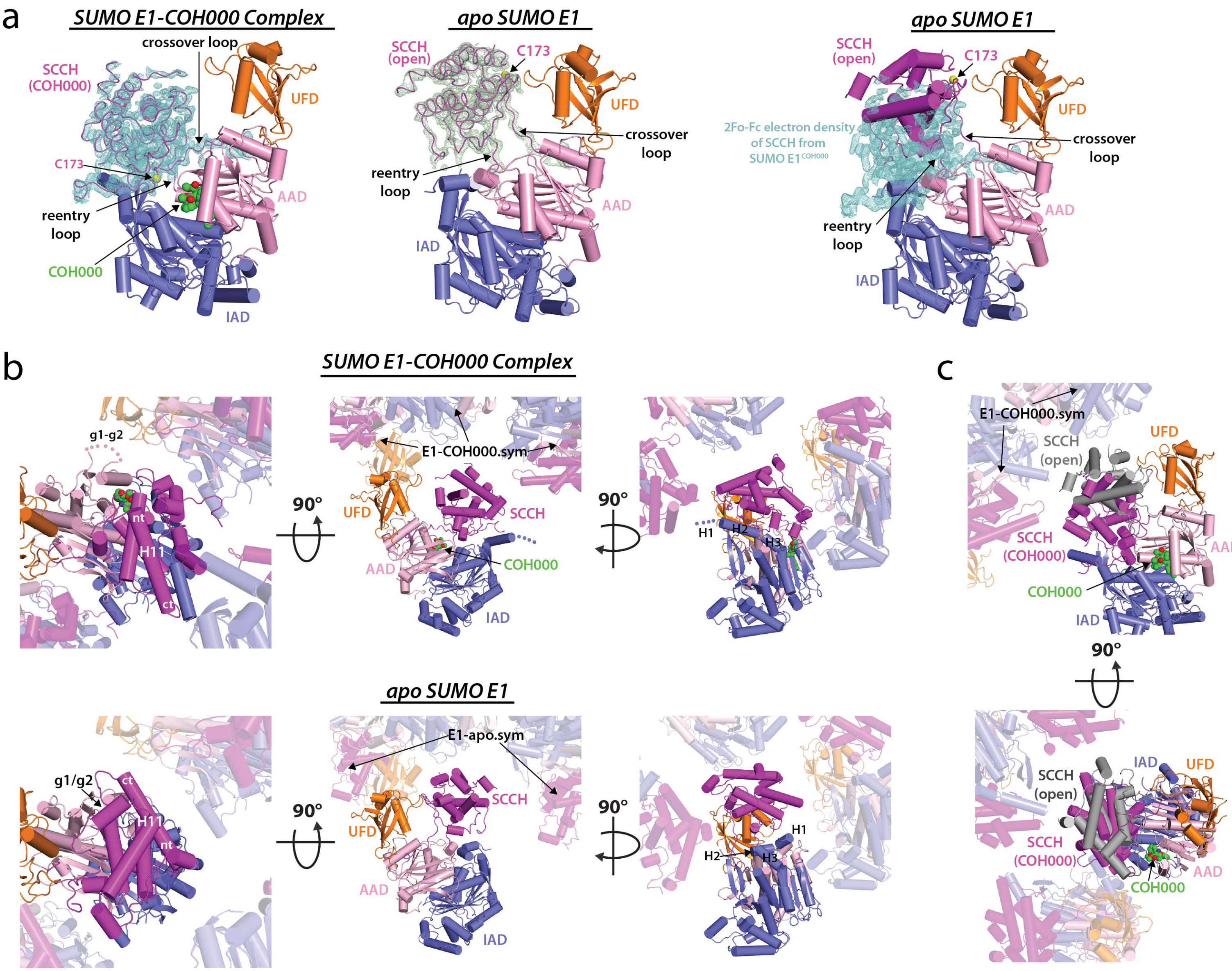

Supplementary Figure 1 | Comparison of SUMO E1^{COH000} and SUMO E1^{apo} SCCH domains (related to Figure 1).
(a) Composite omit 2Fo-Fc electron density (contoured at 1σ) is shown (*left*) as cyan mesh for the SCCH domain of the SUMO E1^{COH000} structure and (*middle*) as green mesh for the SCCH domain of the SUMO E1^{apo} structure. SUMO E1 is presented as in ***Fig. 1a***. In the SUMO E1^{COH000} structure, COH000 is shown as spheres with carbons (green), oxygens (red), and nitrogens (blue) colored. (*right*) The electron density (cyan mesh) of the SCCH domain of the SUMO E1^{COH000} structure is superimposed onto the SUMO E1^{apo} structure to indicate the significant rotation of this domain. (b) The (*top row*) SUMO E1^{COH000} and (*bottom row*) SUMO E1^{apo} structures are shown as cartoon representations, with the domains colored and labeled as in (a). In the SUMO E1^{COH000} structure, COH000 is shown as in (a). Symmetry mates within 10 Å of the SCCH domain of each structure are labeled as E1-COH000.sym or E1-apo.sym and are shown as semi-transparent cartoon representations, with the domains colored as in (a). Top (*left*), front (*middle*), and side (*right*) views for both structures are shown for clarity. In the top view of the SUMO E1^{COH000} structure, the disordered g1/g2 region of the AAD is represented by semitransparent pink circles, and the corresponding region in the SUMO E1^{apo} structure is indicated with an arrow. For both structures, SCCH H11 is labeled and its *N*- and *C*-termini are indicated by 'nt' and 'ct', respectively. In the side view, IAD H1, H2, and H3 are labeled. The disordered H1 of the SUMO E1^{COH000} structure is represented by semitransparent blue circles. (c) The SUMO E1^{COH000} structure is shown as cartoon representation and is labeled and colored as in (a). Neighboring symmetry mates are labeled and colored as in (b). Back (*top*) and side (*bottom*) views are shown for clarity. The SCCH domain of the SUMO E1^{apo} structure is shown as a gray cartoon representation and is superimposed onto the SUMO E1^{COH000} structure to show that the neighboring symmetry mates are not close enough to influence the significant domain rotation observed in the SUMO E1^{COH000} structure.

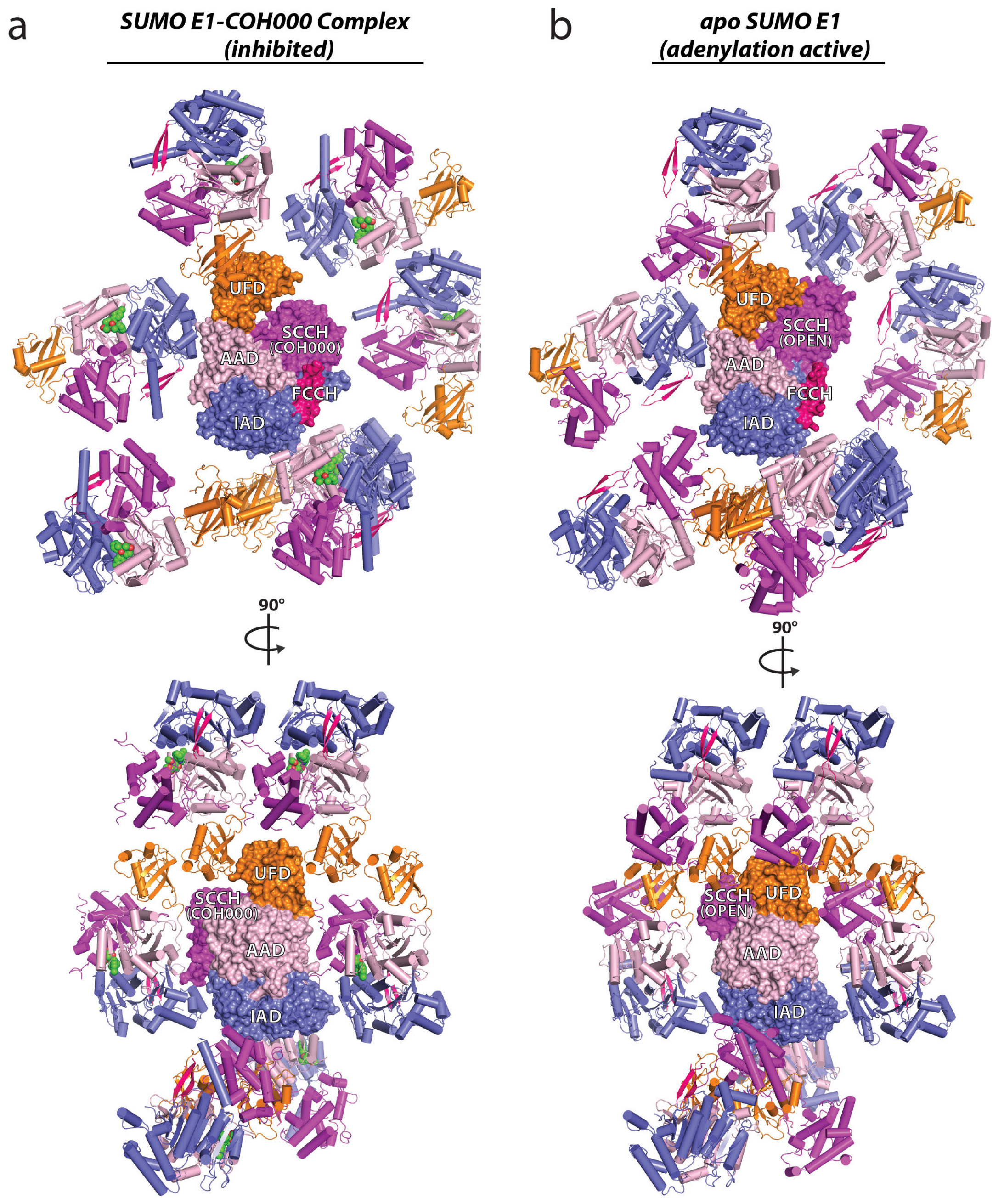

Supplementary Figure 2 | Crystal packing of the SUMO E1^{COH000} and SUMO E1^{APO} structures (related to Figure 1).
SUMO E1 moleclues comprising the crystallographic asymmetric units of the SUMO E1^{COH000} (a) and SUMO E1^{apo} (b) structures are shown as surface representations in the same orientation with domains colored and labeled as in ***Fig 1a***. In the SUMO E1^{COH000} structure, COH000 is shown as spheres. Symmetry-related molecules within 10 Å of each structure are shown as cartoon representations, with the domains colored as above. Two views of the structures related by a 90 degree rotation about the vertical axis are presented.

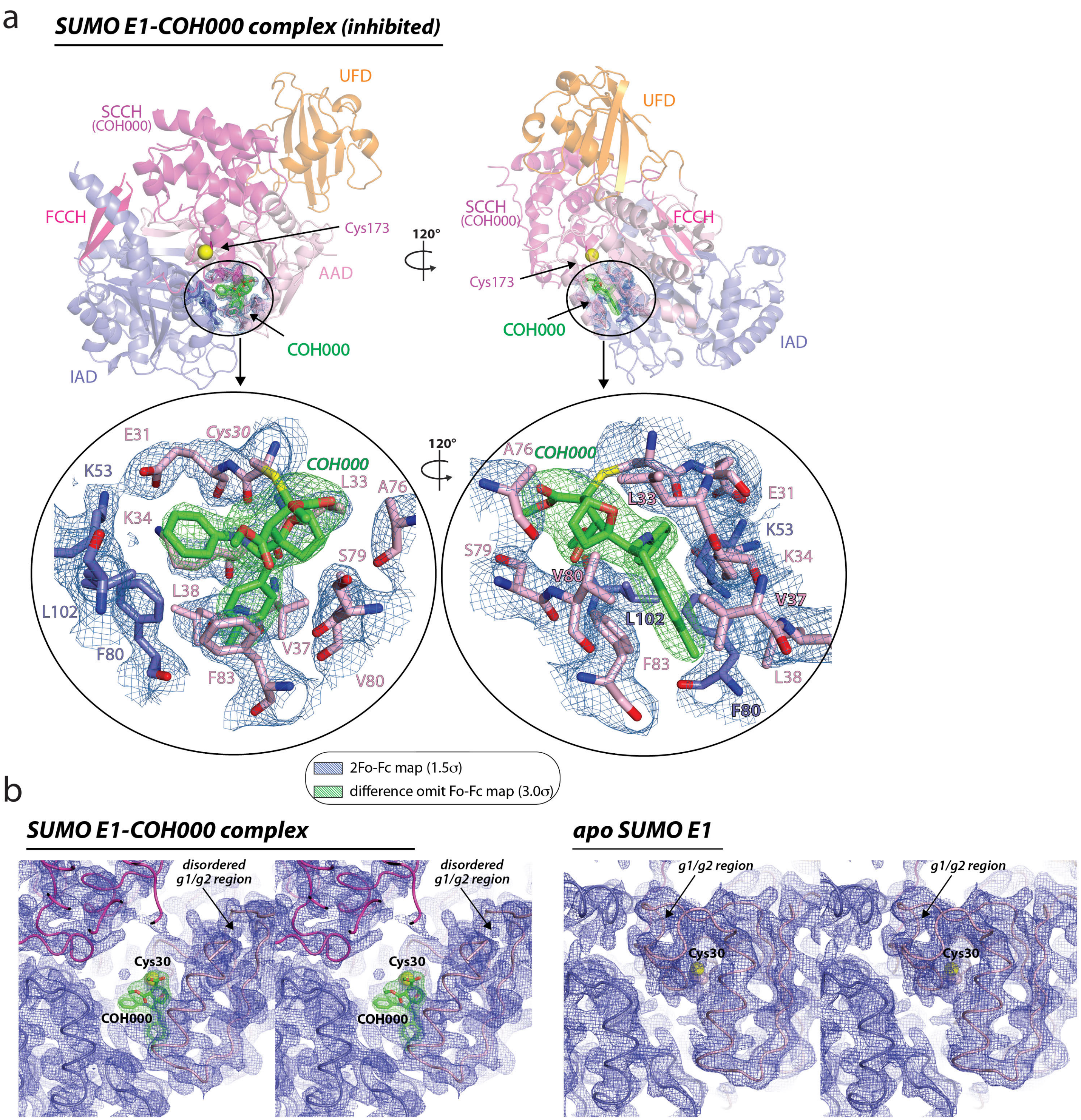

Supplementary Figure 3 | Electron density of the SUMO E1-COH000 interface (related to Figure 2).
(a) COH000 and the COH000 binding site of SUMO E1 from the SUMO E1^{COH000} crystal structure are shown as stick representations, colored as in ***Fig. 1a***. 2Fo-Fc electron density (contoured at 1.5σ) for SUMO E1 residues contacting COH000 is shown as blue mesh. Fo-Fc omit difference electron density for COH000 (contoured at 3.0σ) is shown as green mesh. Two views of the interface related by a 120 degree rotation about the vertical axis are presented. (b) Stereoview of the 2Fo-Fc electron density map (contoured at 1.0σ) for the cryptic COH000 binding site of SUMO E1 is shown for the SUMO E1-COH000 complex (left) and the apo SUMO E1 structure (right). In the apo SUMO E1 structure the g1/g2 region is very well ordered and covers the COH000 binding site. In the SUMO E1-COH000 complex structure the g1/g2 region is fully disordered, allowing COH000 access to its binding pocket.

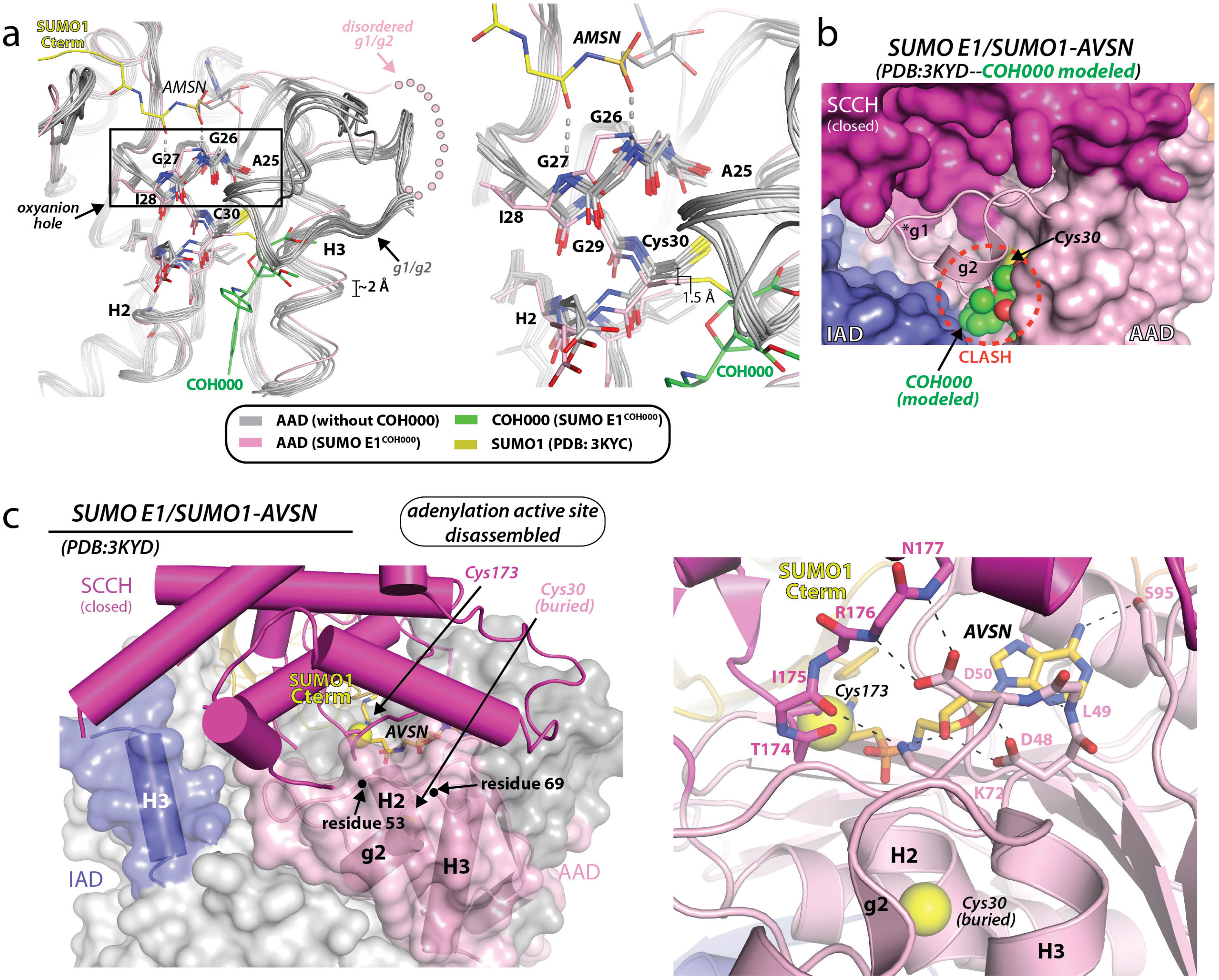

Supplementary Figure 4 | COH000-binding disrupts the SUMO E1 active site (related to Figure 3).
(a) (*left*) The AADs of the SUMO E1^{COH000} structure (pink) and other SUMO E1 structures (PDB: 3KYC, 3KYD, 1Y8Q_Chain B, 1Y8Q_Chain D, 1Y8R_Chain B, 1Y8R_Chain D; gray) were superimposed using residues 15-45 and 88-92 of the AAD for alignment and are shown as tubes. The N-terminal half of H2^{AAD} is shown as sticks and the oxyanion hole is boxed. The disordered g1/g2 region of the SUMO E1^{COH000} structure is shown as semitransparent pink circles. The C-terminus of SUMO1 (yellow) and AMSN (gray) are shown as sticks (from PDB: 3KYC). (*right*) Magnified view around the SUMO E1 oxyanion hole. (b) COH000 was docked onto the SUMO E1/SUMO1-AVSN structure (PDB: 3KYD) as in ***Fig. 3a***. SUMO E1 is shown as in ***Fig. 3a***. (c) (*left*) The SUMO E1/SUMO1-AVSN structure is shown in the same orientation as the structures in ***Fig. 3b***. Regions undergoing conformational changes are colored by their domain as in ***Fig. 1a*** and the rest of the structure is colored light gray. Cys173 (catalytic cysteine) and Cys30 are represented with yellow spheres. (*right*) A magnified view of the thioester bond formation active site is shown as cartoon representation. The side chains of key residues in the AAD are shown as sticks and labeled. Residues 53 and 69 are labeled in the left panel to mark the begining and end of the g1/g2 region.

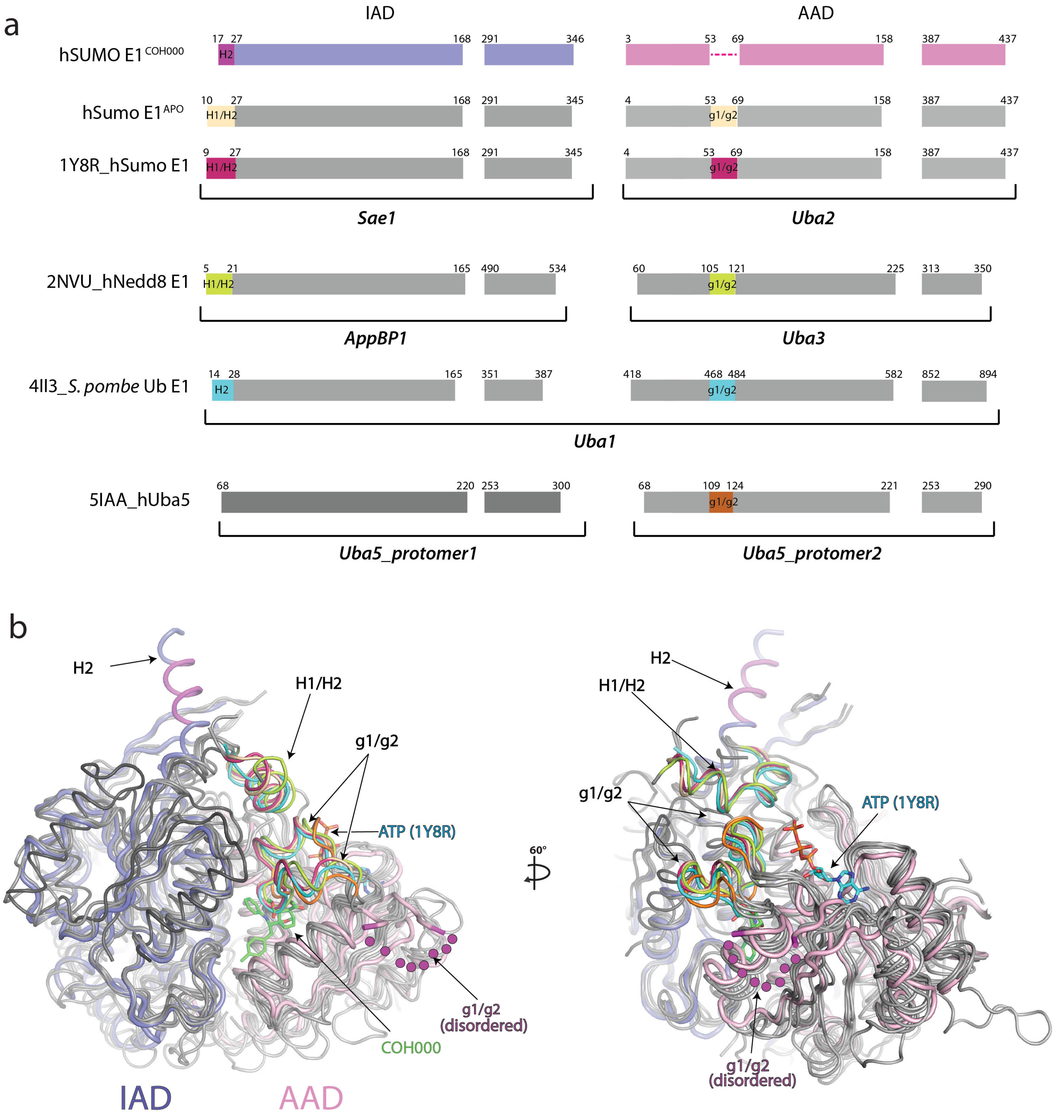

Supplementary Figure 5 | Overall structure alignment of adenylation domains of Ubl E1s (related to Figure 5).
(a) Schematic representation of adenylation domains of a panel of Ubl E1 structures. N-terminal helices H1/H2 from IAD domain and g1/g2 region from AAD domain are highlighted and color-coded. The two subunits of the SUMO E1 and Nedd8 E1 heterodimers are labeled accordingly, Ub E1 is a single polypeptide. Uba5 is a homodimer with two AADs and no IAD. The PDBs used to highlight the structural elements are indicated to the left of the schematic. (b) The SUMO E1^{COH000} along with other E1s structures are shown as coil representations, with the domains colored as in (a).

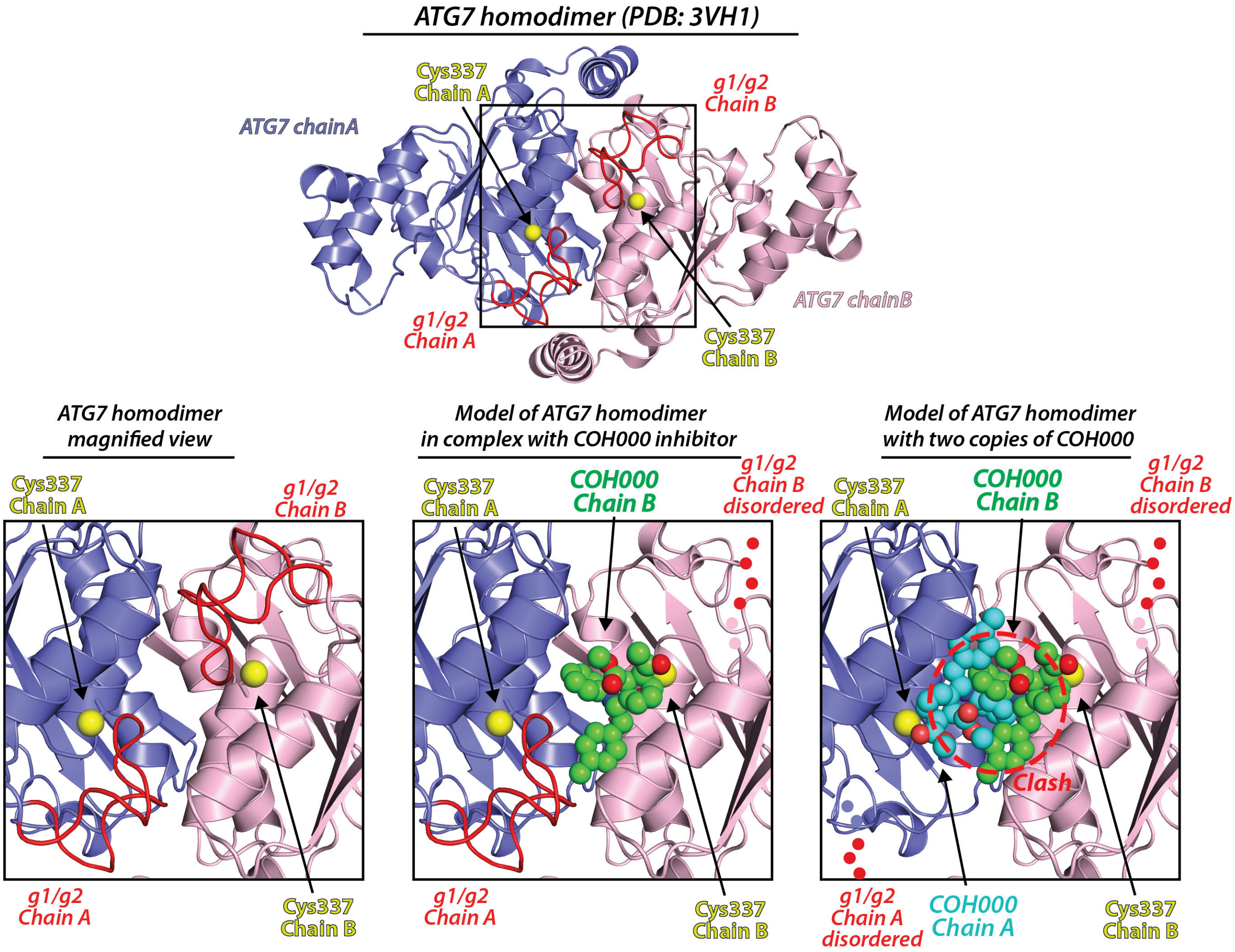

Supplementary Figure 6 | Model of COH000 binding to ATG7 homodimer (related to Figure 5).
(*top*) The ATG7 homodimer was generated using the asymmetric unit and neighboring symmetry mate of the ATG7 structure (PDB: 3VH1) and is shown as cartoon representation. The regions that correspond to g1/g2 in the SUMO E1 structures are colored red. Cys337, which corresponds to Cys30 in SUMO E1, is shown as a yellow sphere in both chains. (*bottom left*) Magnified view of the region containing Cys337 and g1/g2 from the ATG7 homodimer. (*bottom middle*) Model of COH000 binding to Cys337 of chain B of the ATG7 homodimer. The g1/g2 region from chain A is part of the binding pocket while the g1/g2 region of chain B is disordered. (*bottom right*) If COH000 binds to Cys337 of both chains in the ATG7 homodimer, both g1/g2 regions would be disordered and the two copies of the compound would clash with each other.

SUPPLEMENTARY NOTE

COH000 has the IUPAC chemical name of dimethyl 4-[1-anilino-2-(4-methylphenyl)ethyl]-7-oxabicyclo[2.2.1]hepta-2,5-diene-2,3-dicarboxylate. COH000 was prepared and used as a racemic mixture (single diastereomer relevant to the exocyclic stereo center highlighted below).

Chemical synthesis and characterization of COH000

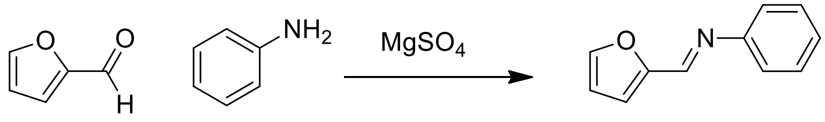

A round bottom flask was charged with furan-2-carbaldehyde (5 g, 52.0 mmol), $MgSO_4$ (6.58 g, 54.6 mmol) and dichloromethane (52 ml) at room temperature. To this solution was then added aniline (5.70 mL, 62.4 mmol) and the resulting mixture was stirred overnight, becoming a pink color. Upon completion, the $MgSO_4$ was removed via filtration and the filtrate was concentrated in vacuo to give a dark oil that was used without further purification.

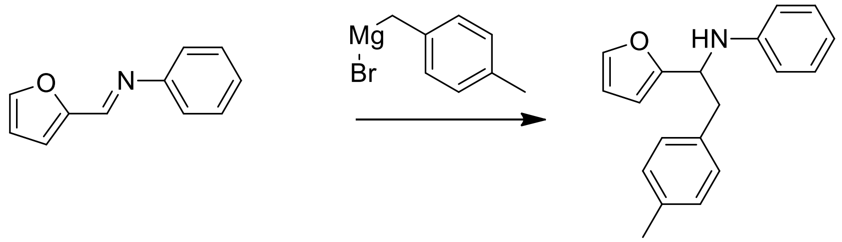

A flame-dried round bottom flask was charged with (E)-N-(furan-2-ylmethylene)aniline (material from above, ~2.9 g) and anhydrous ethyl ether (100 ml) at room temperature. To this solution was then added (4-methylbenzyl)magnesium bromide (0.5 M in THF, 51 ml, 25.5 mmol) and the combined solution was stirred overnight. LCMS analysis indicated that the reaction was complete after 19 h. The solution was poured into saturated ammonium chloride and extracted twice with EtOAc. The organic fraction was then dried over sodium sulfate and concentrated in vacuo to give a yellow oil. Purification on silica gel (50 g biotage column, 1%-5% hexane/EtOAc eluent) gave N-(1-(furan-2-yl)-2-(p-tolyl)ethyl)aniline (1.81 g) as a yellow oil.

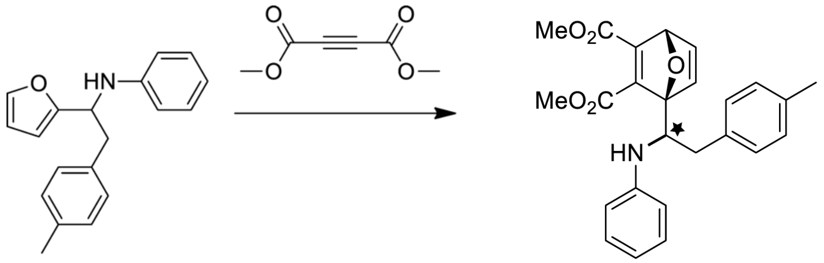

COH000

A round bottom flask was charged with N-(1-(furan-2-yl)-2-(p-tolyl)ethyl)aniline (3.2 g, 11.5 mmol) and toluene (60 ml) and heated to reflux under an atmosphere of nitrogen. To the refluxing solution was then added dimethyl acetylenedicarboxylate (2.55 ml, 20.7 mmol) dropwise and the heating was continued for 3 h. The reaction mixture was continued to reflux for 24 hrs after which it was cooled and the solvent was removed on the rotary evaporator. The resulting brown mass containing the product as a diastereomeric mixture (52:48), with the single diastereomer relevant to the exocyclic stereo center highlighted by a black star above, was

purified via centrifugal thin layer chromatography using a Chromatotron™ and 20% ethyl ether in hexanes as an eluent mixture and was clean by HPLC and its identity confirmed by NMR (666 mg).

Characterization:
^{1}H-NMR ($CDCl_3$, 500 MHz) δ 7.11 (dd, *J* = 15.9, 7.9 Hz, 4H), 7.07 – 6.97 (m, 3H), 6.71 (t, *J* = 7.3 Hz, 1H), 6.61 (d, *J* = 8.0 Hz, 2H), 6.50 (d, *J* = 5.3 Hz, 1H), 5.74 (d, *J* = 1.9 Hz, 1H), 4.59 (dd, *J* = 7.8, 5.9 Hz, 1H), 3.75 (s, 3H), 3.42 (s, 3H), 2.98 (dd, *J* = 13.9, 5.9 Hz, 1H), 2.91 (dd, *J* = 14.0, 7.8 Hz, 1H), 2.29 (s, 3H). ^{13}C-NMR ($CDCl_3$, 125 MHz) δ 165.03, 162.74, 156.70, 149.85, 146.68, 143.42, 143.07, 136.07, 135.11, 129.88, 129.31, 129.14, 118.01, 113.46, 102.16, 83.97, 53.37, 52.43, 52.33, 37.38, 21.27. The NMR spectra are shown below that demonstrates that the compound has correct number of hydrogen and carbon atoms and chemical structure. HRMS for $C_{25}H_{25}NO_5$ [M+H] expected = 420.1805, found = 420.1794.

^{1}H-NMR Spectrum of COH000

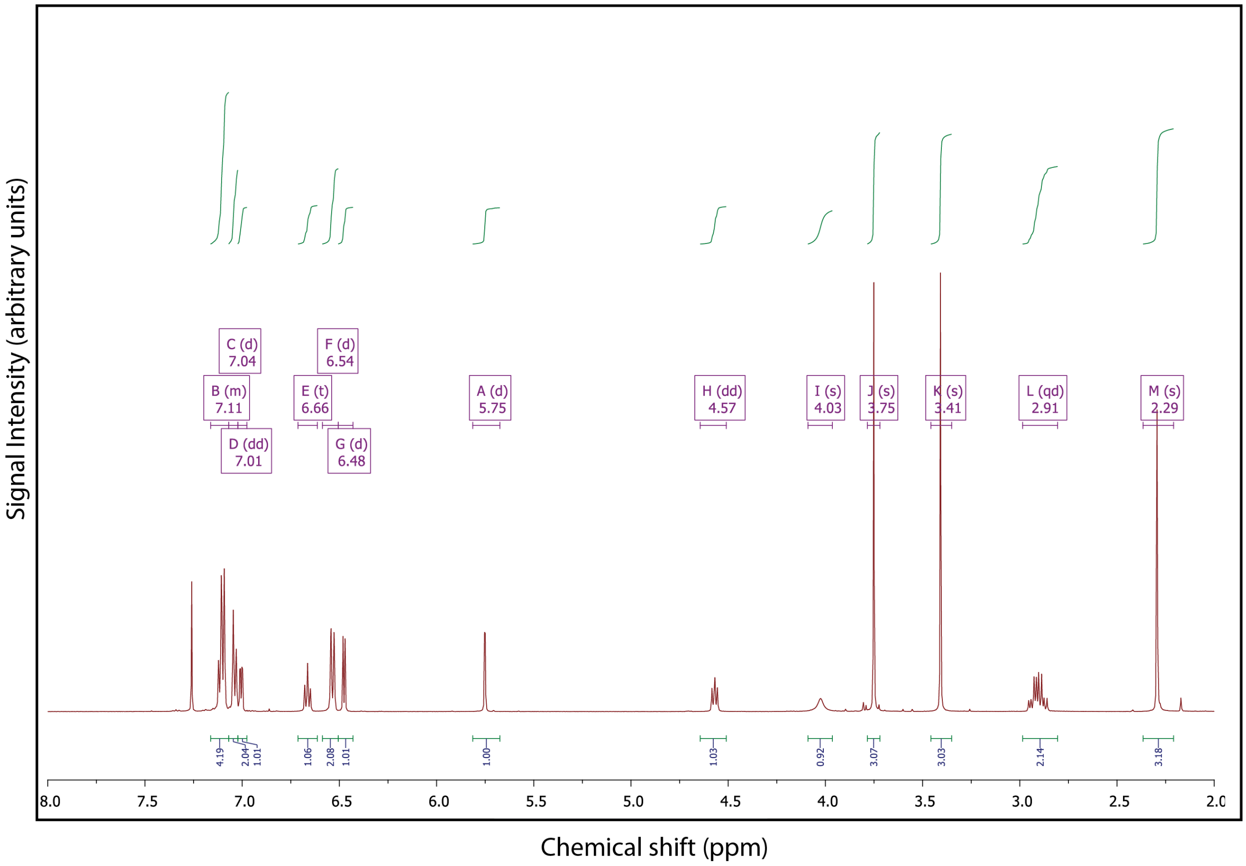

^{13}C-NMR Spectrum of COH000

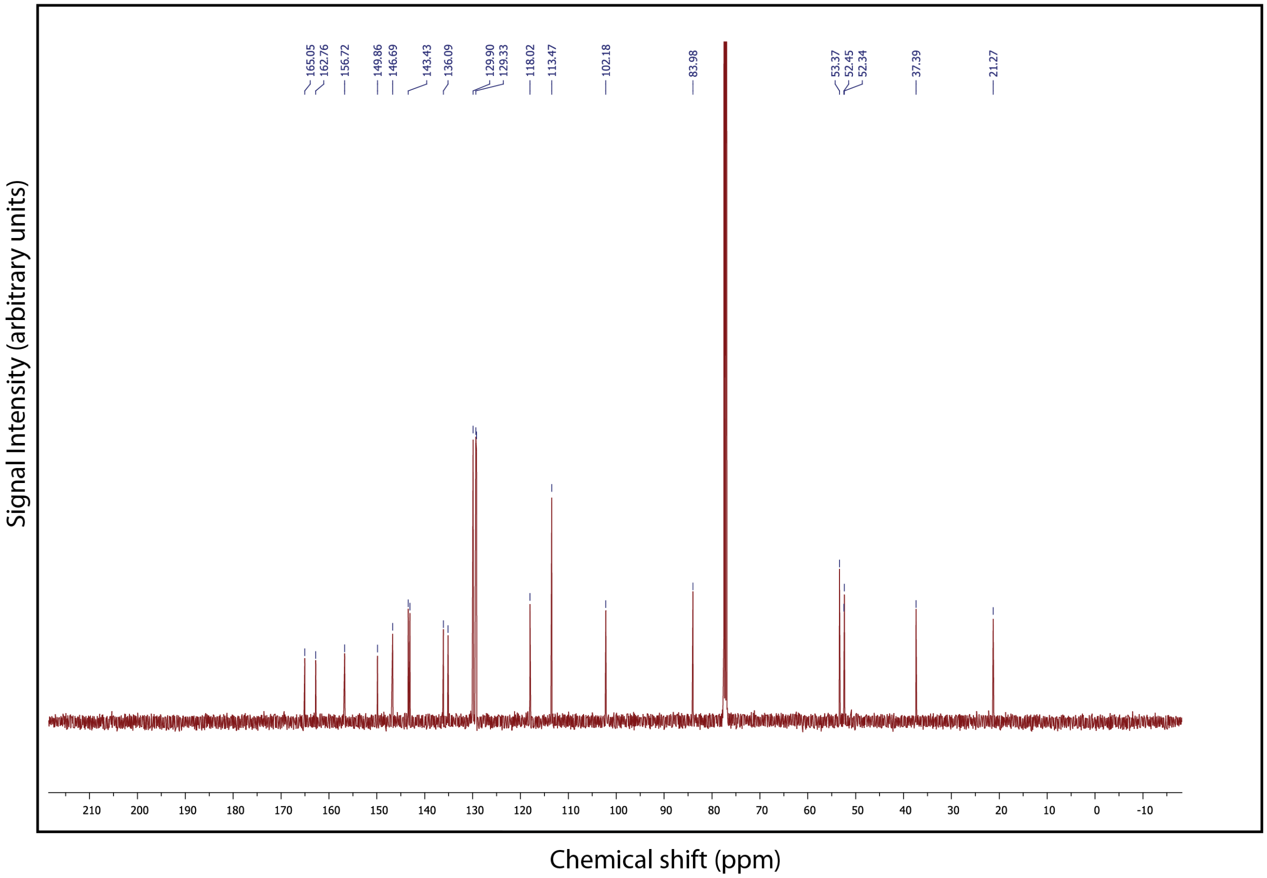

Supplement: Supplementary file 1 — Supplementary Information [file 41467_2018_7015_MOESM1_ESM.pdf]
