# Supplementary material for: Molecular mechanism of a covalent allosteric inhibitor of SUMO E1 activating enzyme
Source: Nat Commun. 2018 Dec 4;9:5145. doi: 10.1038/s41467-018-07015-1 (PMC6279746; doi:10.1038/s41467-018-07015-1)
Supplement: Supplementary file 3 — Description of Additional Supplementary Files [file 41467_2018_7015_MOESM3_ESM.pdf]

Description of Additional Supplementary Files

File Name: Supplementary Movie 1

Description: The movie starts with an overview of the adenylation active SUMO E1 structure (PDB:1Y8R) with E1 shown as a cartoon representation and ATP shown as spheres. AAD elements are colored pink, IAD elements are colored slate, and the SCCH domain is shown in magenta. The view next zooms into the SUMO E1 active site and residues that make direct contacts to ATP and are important for adenylation are shown as sticks. ATP and the SUMO E1 side chains are next hidden, and the g1/g2 region of the SUMO E1 AAD that becomes disordered upon COH000 binding flashes on and off. COH000 (shown as spheres) next floats to its SUMO E1 binding site and the g1/g2 region disappears to represent disorder. Cys30 of the SUMO E1 AAD which forms a covalent adduct with COH000 is shown as a yellow sphere. The view next zooms back out to an overview of the structure, and elements of the IAD that become displaced from the active site in the SUMO E1COH000 crystal structure flash on and off. This is followed by morphing of the SCCH domain and IAD elements from the open adenylation active conformation (PDB:1Y8R) to the COH000-inhibited conformation observed in the SUMO $E1^{COH000}$ reported in this study (PDB:6CWY). This shows the 180- degree rotation of the SCCH domain, displacement of H2 from the E1 active site, and formation of a new SCCH domain IAD/AAD interface in the COH000-inhibited conformation. This is followed by a transition back to the open adenylation active conformation and a 180-degree rotation of the structure so that the viewer can observe the transition from the open adenylation active conformation to the COH000-inhibited conformation from the opposite side of the E1.
